# Combinations of Genetic Data Present in Bipolar Patients, but Absent in Control Persons

**DOI:** 10.1371/journal.pone.0143432

**Published:** 2015-11-20

**Authors:** Erling Mellerup, Ole A. Andreassen, Bente Bennike, Henrik Dam, Srdjan Djurovic, Thomas Hansen, Martin Balslev Jorgensen, Lars Vedel Kessing, Pernille Koefoed, Ingrid Melle, Ole Mors, Thomas Werge, Gert Lykke Moeller

**Affiliations:** 1 Laboratory of Neuropsychiatry, Department of Neuroscience and Pharmacology, University of Copenhagen, Blegdamsvej 9 O-6102, DK-2100 Copenhagen, Denmark; 2 Psychiatric Centre Copenhagen, Department O, Copenhagen University Hospital, Rigshospitalet, Blegdamsvej 9 O-6102, DK-2100 Copenhagen, Denmark; 3 Department of Psychiatry, Oslo University Hospital and Institute of Psychiatry, University of Oslo, Kirkeveien 166. 0407 Oslo, Norway; 4 Department of Medical Genetics, Oslo University Hospital and Institute of Psychiatry, University of Oslo, Kirkeveien 166. 0407 Oslo, Norway; 5 Department of Biological Psychiatry, Mental Health Centre Sct. Hans, Copenhagen University Hospital, Boserupvej 2, DK-4000 Roskilde, Denmark; 6 Centre for Psyciatric Research, Aarhus University Hospital, Skovagervej 2, DK-8240 Risskov, Denmark; 7 Genokey ApS, ScionDTU, Technical University Denmark, Agern Allé 3, DK-2970 Hoersholm, Denmark; University of Frankfurt, GERMANY

## Abstract

The main objective of the study was to find combinations of genetic variants significantly associated with bipolar disorder. In a previous study of bipolar disorder, combinations of three single nucleotide polymorphism (SNP) genotypes taken from 803 SNPs were analyzed, and four clusters of combinations were found to be significantly associated with bipolar disorder. In the present study, combinations of four SNP genotypes taken from the same 803 SNPs were analyzed, and one cluster of combinations was found to be significantly associated with bipolar disorder. Combinations from the new cluster and from the four previous clusters were identified in the genomes of 209 of the 607 patients in the study whereas none of the 1355 control participants had any of these combinations in their genome.

## Introduction

In a previous study of bipolar disorder, we analyzed 803 SNPs belonging to genes related to signal transmission in the brain [1]. Combinations of three SNP genotypes taken from the 803 SNPs were counted; the theoretical number of such combinations is 2,321,319,627, and we found that the 1355 control persons and 607 patients contained 1,985,613,130 combinations. Of these, 1,719,002,329 were common for the two groups; 208,699,590 were found in control participants only; and 57,911,211 combinations were found exclusively in patients. Among these last combinations, four clusters, each containing about 40 combinations, were found to be significantly associated with bipolar disorder. The four clusters corresponded to four genetic subgroups of patients, one of which was significantly different from the three others with respect to number of manic and depressive episodes [2]. Combinations from the four clusters were present in the genome in 156 of the 607 patients whereas none of the 1355 control participants had such a combination in their genome. For the present study, we used the data from the previous work [1] to answer two questions that computational limitations left us unable to address in the initial analyses.

The first question is whether combinations of four SNP genotypes taken from the same 803 SNPs can reveal more clusters of combinations associated with bipolar disorder? Because the theoretical number of combinations of four SNP genotypes is 1,392,791,776,200, the computational challenge remains considerable, and here we report a way to give a partial answer to this question.

The second question is whether some of the 208,699,590 combinations of three SNP genotypes found exclusively in control participants can be considered as protective or risk reducing regarding bipolar disorder?

## Materials and Methods

### SNP genotype data

The Norwegian Scientific-Ethical Committees, the Norwegian Data Protection Agency, the Danish Scientific Committees, and the Danish Data Protection Agency approved the study. All patients gave written informed consent prior to inclusion in the project. Patients, genes, SNP selection, and genotyping have previously been described in detail [1].

### Combinations

Screening for all combinations of two SNP genotypes and all combinations of three SNP genotypes taken from 803 SNPs were performed by array-based mathematical methods where data are represented geometrically, hereby facilitating ultrafast parallel processing [1]. However, due to computational limitations it was not possible to screen for all combinations of four SNP genotypes. Instead a selection of combinations of four SNP genotypes was analyzed. The selection was based on chi square tests starting with the single 803x3 SNP genotypes. Those SNP genotypes that were found to be significantly associated to bipolar disorder with p values <0.05 (without Bonferroni correction) were selected. Only the selected SNP genotypes were combined with all 803x3 SNP genotypes resulting in combinations of two SNP genotypes. Among these combinations those that were significantly associated to bipolar disorder with p values <0.05 were selected. Only the selected combinations of two SNP genotypes were combined with all 803x3 SNP genotypes resulting in combinations of three SNP genotypes. Among these combinations those that were significantly associated to bipolar disorder with p values <0.05 were selected. Only the selected combinations of three SNP genotypes were combined with all 803x3 SNP genotypes resulting in combinations of four SNP genotypes.

From the combinations of four SNP genotypes those occurring exclusively in patients were extracted, in order to analyze whether some of them were significantly associated with bipolar disorder. A combination common for many patients is more likely to be associated to bipolar patients than a combination common for few patients, so permutation tests were performed for combinations of four SNP genotypes that were found in 15, 16 or17 patients. (No combination was common for 18 or more patients). The permutation tests showed that no combination of four SNP genotypes was significantly associated with bipolar disorder.

### Clusters of combinations

A cluster is defined as a group of combinations where all the combinations contain at least one SNP genotype which is the same in all the combinations. The combinations of four SNP genotypes found exclusively in patients were organized into such clusters. The patients belonging to a cluster are those that in their genome have one or more of the combinations in the cluster. As a cluster with many patients is more likely associated to bipolar disorder than a cluster with few patients, permutation tests were performed on clusters with many patients.

### Statistics

Chi square tests were used as a tool to obtain a selection of combinations of four SNP genotypes small enough to be handled by the methods used to analyze combinations [1]. From the selection of combinations of four SNP genotypes, those occurring exclusively in patients were extracted and the association of these combinations with bipolar disorder was tested by permutation tests. Also the association of clusters of these combinations with bipolar disorder was tested by permutation tests. The null hypothesis is that no difference exists between control persons and bipolar patients with respect to the occurrence of combinations of four SNP genotypes, which in the present study meant that all combinations or clusters of combinations found exclusively in patients should be random findings. In permutation tests this means that the labels control and patient shall be interchangeable. If the null hypothesis is true randomly shuffling of the labels will make data sets that look like the real data set. Thus the labels, control and patient, were rearranged creating 1355 pseudo controls and 607 pseudo patients from which combinations of four SNP genotypes, and clusters of combinations were found as described above. This procedure was performed 1000 times. It was counted among the 1000 permutations how often combinations of four SNP genotypes found exclusively in the pseudo patients were common for at least 15, 16 or 17 pseudo patients. If such a result was found more than 50 times in the 1000 permutations the p value is greater than 0,05.

The highest number of patients belonging to a cluster was 73 in the original data set. In 1000 permuted data sets it was counted how often clusters with 73 or more pseudo patients were found. If such clusters were found N times, the p value is N/1000.

### Combinations of three SNP genotypes found exclusively in control persons

All the 208,699,590 combinations of three SNP genotypes found exclusively in control persons were analyzed by the same methods as those used to analyze combinations of three SNP genotypes found exclusively in patients [1].

## Results

Theoretically the number of combinations of four SNP genotypes taken from 803 SNPs is 803!/4!(803–4)! × 3^4^ = 1,392,791,776,200. Because of the computational burden, it was not possible to analyze how many of these combinations were present in the 1355 controls and 607 bipolar patients. Instead a selection of combinations of four SNP genotypes was analyzed. The selection was based on statistics as described in Materials and Methods. From the selected group of combinations of four SNP genotypes, those that occurred exclusively in patients were extracted. Using permutation tests it was found that none of these combinations were significantly associated with bipolar disorder. However, among these combinations, a cluster containing 16 combinations was found to be significantly associated with bipolar disorder (p = 0.011 using permutation test, 1000 permutations). The 16 combinations are shown in Table 1, which also shows the number of patients having these combinations in their genome. The total number of patients belonging to the cluster was 73, of whom 20 also were found in the four clusters in the previous study [1]. Of the 73 patients, 18 carried one combination from the cluster, 15 carried two combinations, 40 carried between 3 and 9 combinations.

**Table 1:** Cluster containing 16 combinations of four SNP genotypes. SNP1 = YWHAH_rs1049583^2^ is common for all 16 combinations.

SNP2	SNP3	SNP4	Number of patients having the combination
TNC_rsG411456^1^	CNTN1_rs278913^1^	CNTNAP2_rs10272638^1^	17
TNC_rs1411456^1^	NFASC_rs2802853^1^	KCNQ3_rs10092250^0^	15
TNC_rs1411456^1^	CNTN1_rs11179168^1^	NFASC_rs9194^1^	15
TNC_rs1411456^1^	CNTN1_rs1056019^1^	CNTNAP2_rs10272638^1^	15
KCNQ2_rs6062929^0^	NRCAM_rs11974486^1^	MBP_rs8090438^0^	15
KCNQ2_rs6062929^0^	KCNN3_rs7547552^1^	ERBB4_rs707284^0^	15
KCNQ2_rs6062929^0^	MBP_rs12959623^1^	MAG_rs1034597^1^	15
ANK3_rs7906905^1^	SPTBN4_rs11672523^1^	KCNQ2_rs6011841^0^	16
CNTNAP2_rs10238991^1^	CNTN1_rs1056019^1^	KCNC1_rs1012105^0^	16
P2RX7_rs1718119^1^	IMPA2_rs3974759^1^	ANK3_rs10761454^0^	15
CNTN1_rs278913^1^	TNC_rs7035322^1^	CNTNAP2_rs10272638^1^	15
MBP_rs8090438^0^	CNTNAP2_rs2972112^0^	GSK3B_rs2037547^0^	15
KCNN3_rs7547552^1^	CNTN1_rs444927^1^	CNTNAP2_rs10464461^0^	15
CNTN1_rs278913^1^	CNTNAP2_rs17170126^1^	KCNN3_rs6426998^1^	15
SCN2B_SCN4B_rs645530^1^	ATP1A2_rs11585375^0^	NRCAM_rs11974486^1^	15
TNR_rs223982^1^	CNTNAP2_rs10277654^2^	NRG1_rs2466094^1^	15

Superscript indicates genotype, wild type

^0^ homozygote

^1^ heterozygote

^2^ variant homozygote.

The 16 combinations contained 36 SNP genotypes.73 patients out of 607 had one or more of these combinations in their genome.

For all other clusters it was found that similar clusters occurred more than 50 times in the 1000 permutations.

In the previous study [1] 208,699,590 combinations of three SNP genotypes were found exclusively in control persons. A permutation test showed that this number could be a random finding. Also those combinations that were common for many controls could be random findings, because the highest number of controls sharing a combination not present in patients was 40, but such a result or higher than 40 was found more than 700 times in 1000 permutations. Permutation tests also showed that all clusters of combinations of three SNP genotypes found exclusively in controls could be random findings.

## Discussion

The genetic basis for polygenic disorders is combinations of genetic variants, if a disorder shows genetic homogeneity this basis consists of only one combination. If the disorder shows genetic heterogeneity several combinations of genetic variants can be basis for the disorder. A combination of genetic variants that is the basis for a polygenic disorder has never been described, probably because analyses of combinations of genetic variants constitute computational and statistical challenges due to the large number of possible combinations, even with moderate numbers of genetic variants [3]. In the present study the number of combinations of only four SNP genotypes taken from 803 SNPs was more than thousand billions four. In order to find one or more combinations significantly associated with a disorder among many billions of combinations calls for various methods and procedures. Obviously, the problem can be addressed by restriction of the analysis to a small number of genetic variants [4,5]. Technically the problem can be attacked by development of fast data-mining methods [1,6,7], and use of specialized hardware, as multiple graphical processing units, to increase scanning speed [8–10]. Such methods has allowed analysis of combinations of genetic variants in studies of esophageal cancer [11], bipolar disorder [1], neuroblastoma [12], and breast cancer [13].

In order to deal with the large number of combinations the statistical analyses can be restricted to the combinations found exclusively in patients, because the chances of finding combinations significantly related to a disorder may be larger in this group of combinations. A further reduction in the number of the patient specific combinations can be obtained by extraction of those combinations that are common to many patients instead of those that are common to few patients.

However, all these methods cannot handle all the combinations of four SNP genotypes taken from 803 SNPs, not to mention the number of combinations of more than four genetic variants taken from studies where thousands of genetic variants have been analyzed. In the present study we have attacked this problem by the use of a selection procedure where not all, but only some combinations have been analyzed. The principle in the procedure is that the distribution between controls and patients for all the genetic variants are analyzed by chi square tests, and those with a low p value are selected. Instead of calculating all possible combinations of two genetic variants, only combinations where at least one genetic variant has a low p value are looked at. And the same principle is used with combinations of three and four genetic variants. By such stepwise selections it is possible to analyze combinations of two, three and four genetic variants, but the cost is that some combinations associated with bipolar disorder but with p values above the threshold may remain undetected. In large data sets as those from genome wide association studies much lower p values may be used in the selection procedure order to obtain numbers of combinations that are manageable. Also biological criteria could have been used in the selection procedure if, e.g., we had reasons to believe that genes for potassium ion channels were particularly important, combinations containing SNP genotypes from such genes could have been used in the selection procedure.

The use of the selection procedure in the present study resulted in exactly the same four clusters of combinations of three SNP genotypes significantly associated to bipolar disorder, as found in the previous study, where all possible combinations were analyzed [1], showing the usefulness of the procedure. Furthermore, the procedure allowed analysis of combinations of four SNP genotypes. No combination of four SNP genotypes found exclusively in patients was significantly associated bipolar disorder, but one cluster of these combinations was found to be significantly associated to bipolar patients. The cluster (Table 1) contained as little as 16 combinations out of more than thousand billion combinations, but 73 patients had one or more of the 16 combinations in their genome. Of the 73 patients, 20 were also among the 156 patients in the four clusters found in the previous study [1]. Thus, 53 patients could be added to the 156 patients, so that 209 out of 607 patients had some of the combinations from the five clusters in their genome. As none of the 1355 control persons had any of these combinations in their genome each of the five clusters may be viewed as general risk factors, and the accumulation of combinations from the clusters in the genome of the single patient may be seen as personal risk factors for bipolar disorder.

All combinations in a cluster contain a common SNP genotype, and in the cluster found in the present study (Table 1), all 16 combinations contained the variant homozygote of YWHAH_rs1049583. In this respect it is interesting that we have found this variant to be associated with bipolar disorder in a study where the present data together with heterogeneous data from genome-wide association studies, protein–protein interaction screens, disease similarity, linkage studies, and gene expression experiments were combined into a multi-layered evidence network [14].

Protective or risk-reducing genes have been found for some diseases [15–17], raising the question whether some of the more than 200 million combinations of three SNP genotypes found exclusively in control persons [1] were significantly associated with not having bipolar disorder; however, we found no such combinations or clusters of combinations. This result does not exclude that combinations of genetic variants may be protective or risk reducing regarding bipolar disorder; because if such combinations are present in small groups of control persons, the present approach may not have identified them. However, the result indicated that specific combinations of SNP genotypes that are protective or risk reducing with respect to bipolar disorder are not present in the genomes of large groups of control persons.

## Conclusion

It is generally accepted that the genetic basis for polygenic disorders are combinations of genetic variants, but due to the high number of possible combinations it is difficult to find those that are the genetic basis. Using a procedure where only selections of combinations were analyzed it was possible to find clusters of combinations of SNP genotypes significantly associated to bipolar disorder. The combinations in the clusters were present in the genome of 35% of the patients, but absent in all control subjects. This finding indicates a role for combinations of genetic data in clinical work with diagnosis and estimation of risk of disease, but replication studies are warranted in order to further evaluate the relevance of combinations of genetic data. Such studies may be new, but combinations of genetic variants may also be analyzed in studies already published.

## Supporting Information

S1 Table The complete data set. (TXT)

S1 Text Title and legend to S1 Table. (DOCX)

## References

[1] Koefoed P, Andreassen O, Bennike B, Dam H, Djurovic S, Hansen T, et al (2011) Combinations of SNPs related to signal transduction in bipolar disorder. PLoS ONE 6: e23812 10.1371/journal.pone.0023812 21897858 PMC3163586

[2] Mellerup E, Andreassen O, Bennike B, Dam H, Djurovic S, Hansen T, et al (2012) Connection between genetic and clinical data in bipolar disorder. PLoS ONE 7: e44623 10.1371/journal.pone.0044623 23028568 PMC3447882

[3] Jiang X, Neapolitan RE (2012) Mining pure, strict epistatic interactions from high-dimensional datasets: ameliorating the curse of dimensionality. PLoS ONE 7: e46771 10.1371/journal.pone.0046771 23071633 PMC3470561

[4] Emily M, Mailund T, Hein J, Schauser L, Schierup MH (2009) Using biological networks to search for interacting loci in genome-wide association studies. Eur J Hum Genet 17: 1231–1240. 10.1038/ejhg.2009.15 19277065 PMC2986645

[5] Slavin TP, Feng T, Schnell A, Zhu X, Elston RC (2011) Two-marker association tests yield new disease associations for coronary artery disease and hypertension. Hum Genet 130: 725–733. 10.1007/s00439-011-1009-6 21626137 PMC3576836

[6] Yee J, Kwon MS, Park T, Park M (2013) A modified entropy-based approach for identifying gene-gene interactions in case-control study. PLoS ONE 8: e69321 10.1371/journal.pone.0069321 23874943 PMC3715501

[7] Goudey B, Rawlinson D, Wang Q, Shi F. Ferra H, Campbell RM, et al (2013) GWIS-model-free, fast and exhaustive search for epistatic interactions in case-control GWAS. BMC Genomics 14 Suppl 3: S10 10.1186/1471-2164-14-S3-S10 23819779 PMC3665501

[8] Prabhu S, Pe'er I (2012) Ultrafast genome-wide scan for SNP-SNP interactions in common complex disease. Genome Res 22: 2230–2240. 10.1101/gr.137885.112 22767386 PMC3483552

[9] Kam-Thong T, Czamara D, Tsuda K, Borgwardt K, Lewis CM, Erhardt-Lehmann A, et al (2010) EPIBLASTER-fast exhaustive two-locus epistasis detection strategy using graphical processing units. Eur J Hum Genet 19: 465–471. 10.1038/ejhg.2010.196 21150885 PMC3060319

[10] Hu X, Liu Q, Zhang Z, Li Z. Wang S, He L, et al (2010) SHEsisEpi, a GPU-enhanced genome-wide SNP–SNP interaction scanning algorithm, efficiently reveals the risk genetic epistasis in bipolar disorder. Cell Res 20: 854–857. 10.1038/cr.2010.68 20502444

[11] Xie Q, Ratnasinghe LD, Hong H, Perkins R, Tang ZZ, Hu N, et al (2005) Decision forest analysis of 61 single nucleotide polymorphisms in a case-control study of esophageal cancer; a novel method. BMC Bioinf 6 Suppl 2: S4. 10.1186/1471-2105-6-S2-S4 PMC1637030 16026601

[12] Capasso M, Calabrese FM, Iolascon A, Mellerup E (2014) Combinations of genetic data in a study of neuroblastoma risk genotypes. Cancer Genet 207: 94–97. 10.1016/j.cancergen.2014.02.004 24726319

[13] Milne RL, Herranz J, Michailidou K, Dennis J, Jonathan P, Zamora MP, et al (2014) A large-scale assessment of two-way SNP interactions in breast cancer susceptibility using 46 450 cases and 42 461 controls from the breast cancer association consortium. Hum Mol Genet 23: 1934–1946. 10.1093/hmg/ddt581 24242184 PMC3943524

[14] Pers TH, Hansen NT, Lage K, Koefoed P, Dworzynski P, Miller ML, et al(2011) Meta-analysis of heterogeneous data sources for genome-scale identification of risk genes in complex phenotypes. Genet Epidemiol. 35: 318–32. 10.1002/gepi.20580 21484861

[15] Brunner G, Reitz M, Heinecke A, Lippold A, Berking C, Suter L, et al (2013) A nine-gene signature predicting clinical outcome in cutaneous melanoma. J Cancer Res Clin Oncology 139: 249–58. 10.1007/s00432-012-1322-z PMC11824186 23052696

[16] Hartikainen JM, Tengstrom M, Kosma VM, Kinnula VL, Mannermaa A, Soini Y, et al (2012) Genetic polymorphisms and protein expression of NRF2 and Sulfiredoxin predict survival outcomes in breast cancer. Cancer Res 72:5537–46. 10.1158/0008-5472.CAN-12-1474 22964583

[17] Wu QQ, Wang Y, Senitko M, Meyer C, Wigley WC, Ferguson DA, et al (2011) Bardoxolone methyl (BARD) ameliorates ischemic AKI and increases expression of protective genes Nrf2, PPARgamma, and HO-1. Am J Physiol—Renal Physiol 300: F1180–92. 10.1152/ajprenal.00353.2010 21289052 PMC3094059

