# Supplementary material for: Combinations of Genetic Data Present in Bipolar Patients, but Absent in Control Persons
Source: PLoS One. 2015 Nov 20;10(11):e0143432. doi: 10.1371/journal.pone.0143432 (PMC4654514; doi:10.1371/journal.pone.0143432)
Supplement: S1 Text — (DOCX) [file pone.0143432.s002.docx]

S1 Table shows the complete data set with 607 bipolar patients, 1355 controls and 803 SNPs in a relational table with 1963 rows and 804 columns. “,” is used as separator between the cells.

The first column is ID (patient=0, control=1), while the other columns represent the SNP genotypes (0 = wild type homozygote; 1 = heterozygote; 2 = variant homozygote; 3 = NA)

The first row in the table is a header with the name (gene and SNP rs number) of each column.
